# Incidental Detection of a Non-valvular Papillary Fibroelastoma on Transthoracic Echocardiography in an Elderly Patient Undergoing Preoperative Assessment for Hip Repair

**DOI:** 10.7759/cureus.81032

**Published:** 2025-03-23

**Authors:** Abbas Rachid, Batoul Chaaban, Hassan Bitar, Malek Mohammed, Hasan Kazma

**Affiliations:** 1 Internal Medicine, Lebanese University, Beirut, LBN; 2 Cardiology, Lebanese University, Beirut, LBN; 3 Cardiovascular Disease, Bahman University Hospital, Beirut, LBN

**Keywords:** cardiac imaging modalities, cardiac papillary fibroelastoma, cardiac tumours, pre operative evaluation, preoperative evaluation, valvular lesions

## Abstract

Papillary fibroelastoma (PFE) is a rare benign cardiac tumor that is often detected incidentally. This case report describes a 91-year-old Lebanese female who was admitted for a hip fracture and was found to have a nonvalvular cardiac PFE during preoperative evaluation. The patient had a history of progressive dyspnea, and transthoracic echocardiography revealed a pedunculated mass attached to the chordae of the posterior papillary muscle, causing mid-diastolic obstruction. Given the patient’s clinical presentation and imaging findings, close postoperative monitoring in the cardiac care unit was advised. This case highlights the importance of echocardiographic evaluation in elderly patients with unexplained dyspnea and the need for individualized management strategies for incidentally discovered cardiac tumors.

## Introduction

Primary cardiac tumors are rare, comprising less than 5% of all cardiac neoplasms. Myxomas and lipomas are the most frequently encountered, followed by papillary fibroelastosis, which are benign, low-profile tumors accounting for approximately 8% of primary cardiac tumors. In contrast, most cardiac tumors are metastatic in origin [1,2].

We present the case of a 91-year-old Lebanese woman who was admitted for elective surgery following a hip fracture. Her medical history included dyspnea, orthopnea, and paroxysmal nocturnal dyspnea, none of which had been previously investigated. During her preoperative evaluation, a transthoracic echocardiography (TEE) was performed, revealing a mobile mass attached to the chordae of the posterior papillary muscle. The differential diagnosis included a cardiac tumor, vegetation, or thrombus. However, based on its echogenicity, pedunculated morphology, and characteristic movement, the mass was identified as a rare nonvalvular papillary fibroelastoma (PFE).

## Case presentation

A 91-year-old female with a history of hypertension, no history of coronary artery disease, and diabetes mellitus managed with amlodipine 5 mg daily was admitted to the hospital following a fall that resulted in a left hip fracture. The diagnosis was confirmed via bilateral hip radiography. Laboratory investigations revealed stable creatinine levels compared to the previous year, anemia, and no elevation in inflammatory markers (Table 1). A chest radiograph demonstrated an enlarged cardiac silhouette.

**Table 1:** Laboratory investigation of our patient during hospitalization CRP: C-reactive protein; NT-proBNP, N-terminal prohormone of brain natriuretic peptide

Parameter	Patient value	Reference range
WBC	11,800	4,500-11,000/mm^3^
Hemoglobin	9.92 g/dL	13.5-17.5 g/d
Platelets	217,000	150-400x10^9^/L
Creatinine	1.22 mg/dL	<1 mg/dL
Sodium	138 mEq/L	135-145 mEq/L
Potassium	3.7 mEq/L	3.5-5.2 mEq/L
Chloride	103 mEq/L	95-105 mEq/L
CRP	4 mg/L	<5 mg/L
NT-proBNP	400 pg/mL	<1,800 pg/mL
D-dimer	800 ng/mL	<900 ng/mL according to her age

On admission, the patient was hemodynamically stable, with an oxygen saturation (SpO₂) of 95% on room air. She was neither tachycardic nor tachypneic, and +1 bilateral pitting lower limb edema was noted. Pulmonary auscultation revealed fine crackles in the bilateral lower lung fields. She reported progressive dyspnea over the past several months, occurring with minimal physical activity, along with orthopnea and paroxysmal nocturnal dyspnea. On physical examination, a diastolic murmur at the apex was detected. Her AUBHAS2 score was 4, and the Revised Cardiac Risk Index for Preoperative Risk Assessment was 1. Given these findings, a TTE was performed as part of the preoperative evaluation. Electrocardiography (ECG) showed normal sinus rhythm and Pro-BnP (N-terminal prohormone of brain natriuretic peptide) level was measured at 400pg/mL, which is elevated.

**Figure 1:**
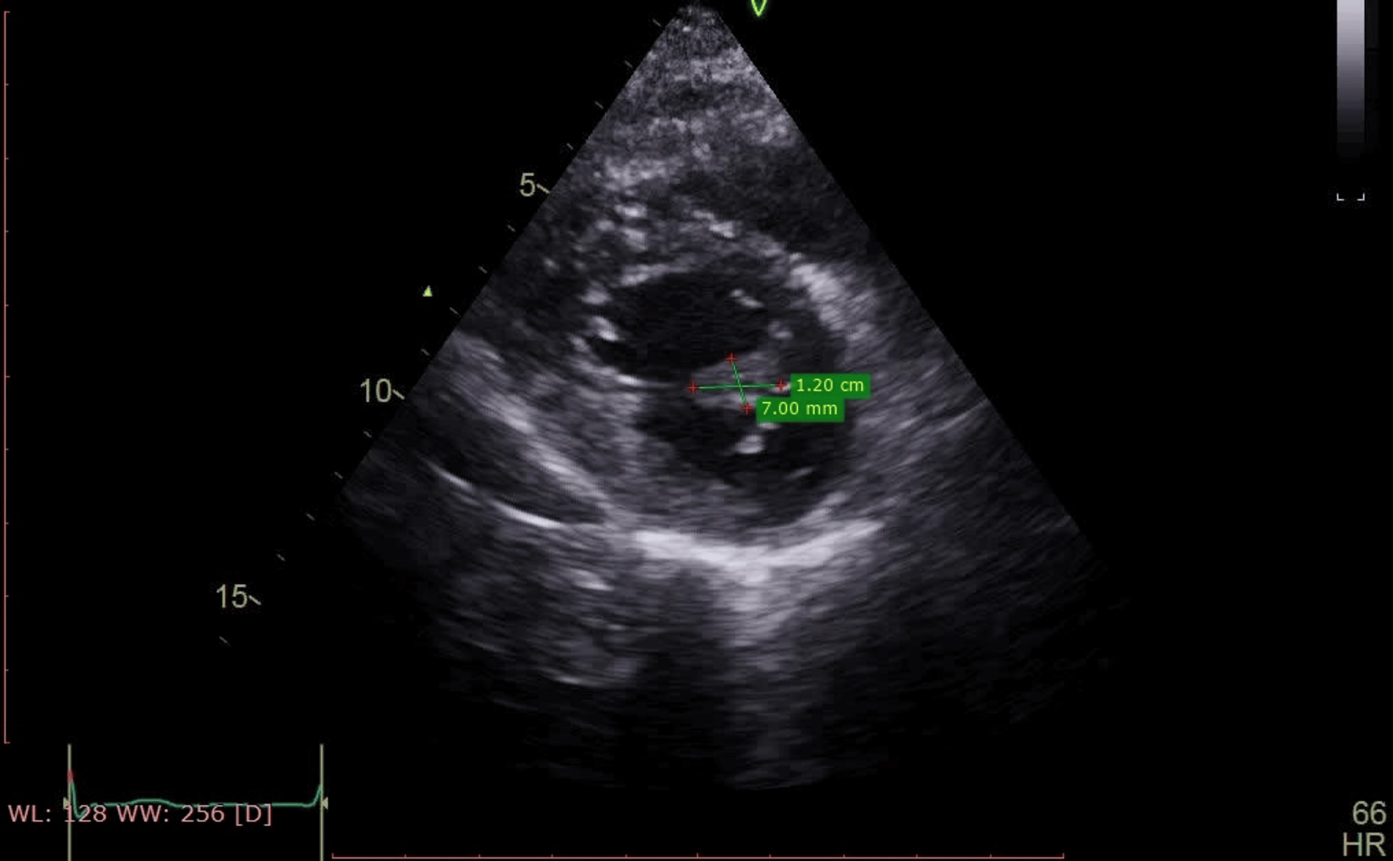
Short-axis view of the non-valvular papillary fibroelastoma of 12 x 7 mm measurement.

**Figure 2:**
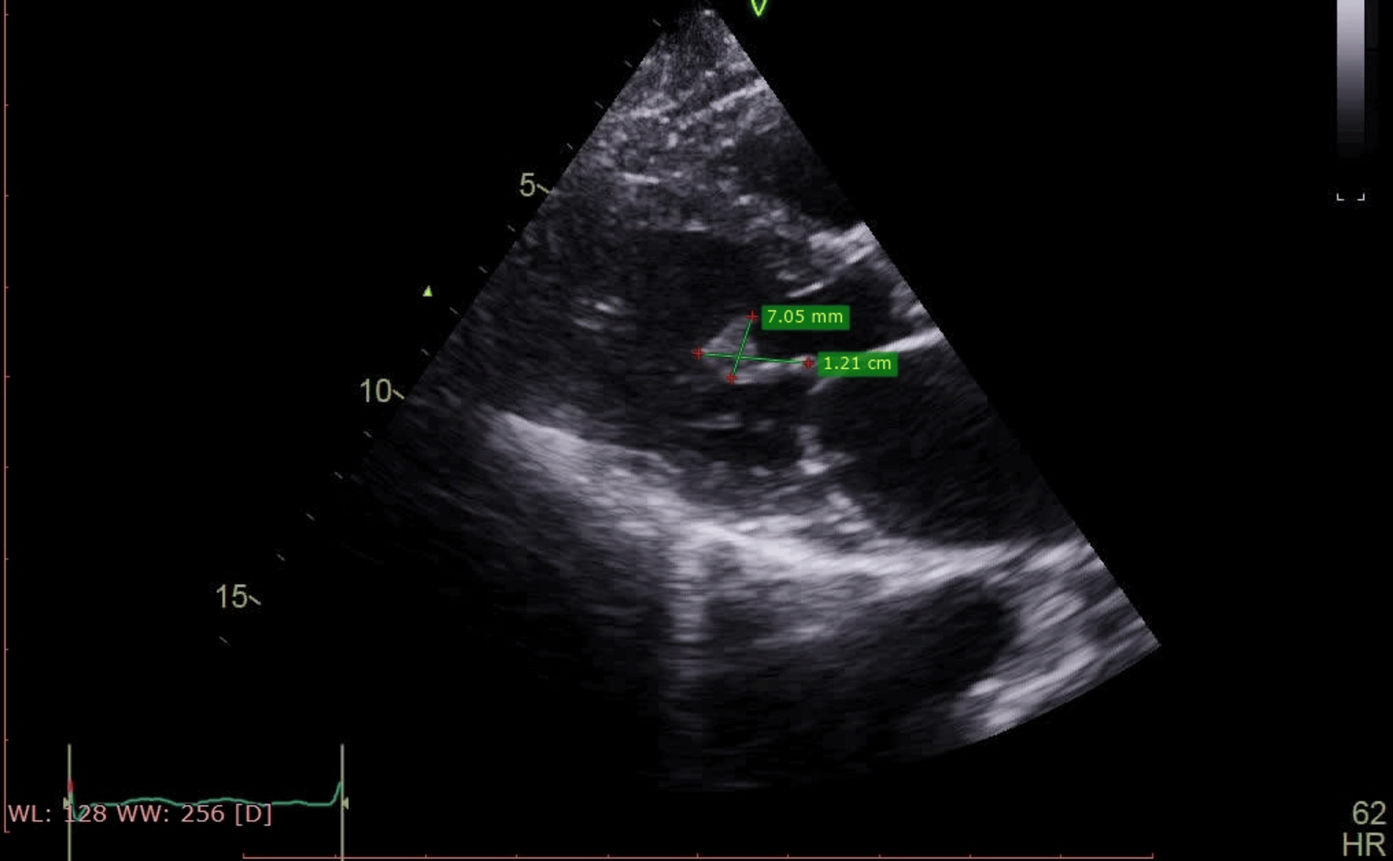
Long-axis view of the non-valvular papillary fibroelastoma of 12 x 7 mm measurement.

**Figure 3:**
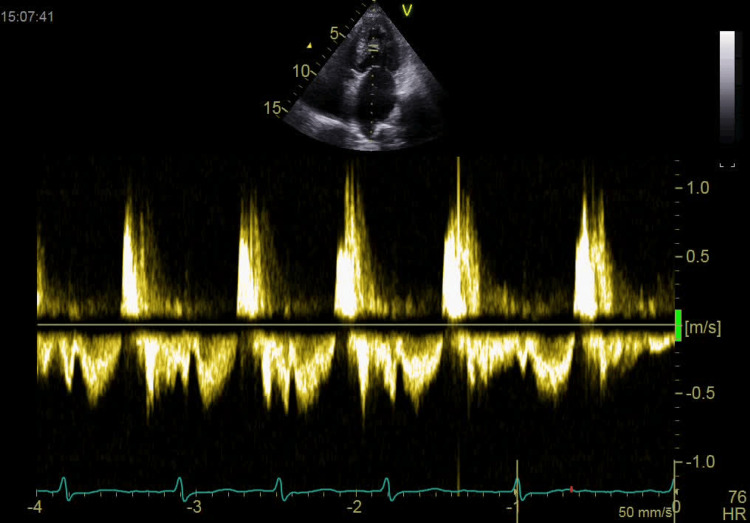
Continuous Doppler of grading diastolic intraventricular obstruction caused by the non-valvular papillary fibroelastoma.

The echocardiographic evaluation revealed a mildly dilated left atrium and a normal-sized left ventricle with fair global contractility. The left ventricular ejection fraction was measured at 56%, with a global longitudinal strain of -12%, indicating elevated left ventricular filling pressure. Both the right atrium and right ventricle were severely dilated, with the right ventricular diameter measuring 44 mm. Additionally, right ventricular systolic function was depressed, as evidenced by a tricuspid annular plane systolic excursion (TAPSE) of 13 mm. The mitral valve exhibited grade 1 regurgitation without stenosis. Notably, a 12 × 7 mm mass, consistent with a PFE, was identified on the chordae of the posterior papillary muscle. This mass caused mid-diastolic obstruction, leading to an intraventricular diastolic gradient of 5.77 mmHg (Figures 1-3).

An echocardiographic assessment of the aortic valve revealed calcification with mild aortic stenosis (AVA by VTI = 1.9 cm²) and grade 1 regurgitation. The pulmonary valve appeared normal. The tricuspid valve exhibited severe regurgitation, with an estimated systolic pulmonary artery pressure (SPAP) of 65 mmHg (45 + 20 mmHg), suggesting pulmonary hypertension primarily due to either a primary cause or chronic embolism. Additionally, the inferior vena cava was dilated to 23 mm, exhibiting less than 50% collapsibility on inspiration. No pericardial effusion was noted. M-mode measurements indicated a left atrial diameter of 3.8 cm, while the left ventricle measured 4.3 cm in end-diastole and 3.1 cm in end-systole.

The patient's D-dimer level was within the normal range for her age, and her Wells score was 1.5 points, indicating a low risk of venous thromboembolism. Further evaluation included a Doppler ultrasound of the peripheral limbs, which ruled out deep vein thrombosis. A CT pulmonary angiogram to exclude chronic pulmonary embolism was not performed due to elevated creatinine levels, and V/Q scan was considered but was not available at the facility. Additionally, cardiac magnetic resonance (CMR) imaging, which could have provided further tissue characterization, was unavailable at the facility. Given the echocardiographic findings of a mobile irregularly shaped mass attached to the chordae of the posterior papillary muscle (Videos 1-3) and clinical presentation, postoperative monitoring in the cardiac care unit was recommended. The patient was initiated on furosemide 40 mg (Lasix) preoperatively for volume management.

**Video 1:** The non-valvular papillary fibroelastoma observed on short-axis TTE view. TTE, transthoracic echocardiography

**Video 2:** The non-valvular papillary fibroelastoma observed on long-axis TTE view. TTE, transthoracic echocardiography

**Video 3:** The non-valvular papillary fibroelastoma observed on four-chamber TTE view. TTE, transthoracic echocardiography

## Discussion

PFE is a rare benign cardiac tumor derived from the endocardium, corresponding to less than 5% to 10% of cases, which corresponds to the third most common primary intracardiac tumor. It usually affects cardiac valves and is rarely related to valve dysfunction. Although rare, fibroelastoma can also involve other locations, including the tendinous cords, endocardium, and papillary muscles, potentially causing mechanical obstruction and an increased risk of embolism [1,2].

Echocardiographically, PFEs are usually seen as small, well-defined, pedunculated masses that primarily affect the valvular endocardium. They are most commonly found on the aortic and mitral valves, as well as the chordae tendineae. Non-valvular occurrences are extremely rare, with an incidence of only 9%, as reported in the series by Gowda et al. [3-5]. The first documented case of PFE was reported in 1975 in the context of an embolic complication leading to myocardial infarction. Since then, PFEs have been recognized as a potential etiology of vascular embolism, stroke, and cardiac arrest. Case reports have linked cardiac papillary fibroelastomas (CPFs) to embolic events affecting the coronary, cerebral, pulmonary, and retinal arteries; however, the precise incidence of such complications remains unclear [3,6]. Our patient presented only with a history of dyspnea due to pulmonary hypertension and diastolic dysfunction with an NYHA score of 2 and no history of embolic event, myocardial infarction, or cerebrovascular accident. Although the NT-ProBNP level was elevated at 400 pg/mL, remaining below the reference threshold, it was still considered high, warranting further evaluation through echocardiography.

On echocardiography, PFEs typically appear as round, oval, or irregularly shaped masses that are well-demarcated and homogeneous in texture. When image quality is optimal, a characteristic "speckled appearance" with peripheral "stippling" may be observed, aiding in their identification. TTE remains the preferred initial diagnostic modality for PFEs due to its non-invasive nature. The sensitivity and specificity of TTE for detecting CPF ≥ 0.2 cm have been reported as 88.9% and 87.8%, respectively, with an overall diagnostic accuracy of 88.4% [3]. Transesophageal echocardiography (TEE) is crucial in identifying PFEs in rare and atypical non-valvular locations and is essential for surgical planning and intraoperative guidance. It is particularly recommended for enhanced visualization of intracardiac structures [7] [8]. The sensitivity of TTE for detecting PFEs smaller than 2 mm is 61.9% compared to 76.6% for TEE [9]. While CMR imaging and computed tomography (CT) provide superior spatial resolution, their routine use is limited by high costs and the need for specialized resources for image acquisition and interpretation, particularly in the case of cardiac MRI. As with other solid tumors, a definitive diagnosis is established through histopathological examination [10].

In our patient, a mass was identified as being adherent to the lateral papillary muscle. Based on its echogenicity and pedunculated morphology, the mass was detected on TTE as a PFE (Videos 1-3). From our perspective based on hemodynamic principles, the fibroelastoma attached to the lateral papillary muscle generated a mild diastolic intraventricular gradient of 5.7 mmHg, potentially increasing left ventricular filling pressure. This, in turn, may have elevated left atrial and pulmonary capillary wedge pressures, contributing to pulmonary hypertension. The resulting hemodynamic burden could have led to tricuspid regurgitation and right ventricular dysfunction (Figure 3).

Management of PFE typically involves either surgical excision or close monitoring. Surgical removal is generally recommended unless contraindicated to achieve complete tumor excision and intracardiac defect reconstruction. Surgical resection is considered curative and effectively prevents the occurrence or recurrence of embolic events. In symptomatic patients who are poor surgical candidates, anticoagulation may be considered to reduce the risk of embolic complications, although randomized controlled trials do not support this approach [5]. For asymptomatic patients, surgical intervention should be considered when the PFE exceeds 9 mm in size, demonstrates high mobility, or exhibits independent motion, as these factors are associated with an increased risk of adverse outcomes [5].

In our case, we carefully evaluated the risks and benefits of surgical intervention. Considering the patient's age, comorbidities, and the absence of previous embolic events, we opted for anticoagulation therapy with apixaban at a dose of 2.5 mg twice daily, tailored to her weight (55 kg) and age. This decision was based on literature suggesting an increased risk of thromboembolism in similar patients, warranting preventive anticoagulation.

## Conclusions

PFE is a rare but clinically significant cardiac tumor that may present asymptomatically or with complications such as embolism or valvular dysfunction. This case underscores the importance of echocardiography in detecting intracardiac masses, particularly in elderly patients with unexplained dyspnea. While surgical excision remains the preferred treatment for symptomatic or high-risk PFEs, conservative management may be appropriate in select cases. Further studies are needed to guide optimal management strategies, particularly in elderly patients with multiple comorbidities.
